# On Vision Transformer Explainability for Personal Protective Equipment Detection: A Qualitative and Quantitative Analysis

**DOI:** 10.3390/jimaging12050195

**Published:** 2026-04-30

**Authors:** Miriam Di Renzo, Filomena Niro, Patrizia Agnello, Marta Petyx, Fabio Martinelli, Mario Cesarelli, Antonella Santone, Francesco Mercaldo

**Affiliations:** 1 Department of Medicine and Health Sciences “Vincenzo Tiberio”, University of Molise, 86100 Campobasso, Italy; 2 Istituto Nazionale per l’Assicurazione Contro gli Infortuni sul Lavoro, 00144 Roma, Italy; p.agnello@inail.it (P.A.); m.petyx@inail.it (M.P.); 3 Institute for High Performance Computing and Networking, National Research Council of Italy (CNR), 87036 Rende, Italy; 4 Department of Engineering, University of Sannio, 82100 Benevento, Italy

**Keywords:** PPE, protective equipment, federated machine learning, explainability, Grad-CAM, attention heatmap, rollout heatmap, CLS heatmap, safety

## Abstract

The safety of workers in industrial settings is ensured through the correct use of Personal Protective Equipment (PPE). The use of such equipment can be monitored using Deep Learning (DL). Federated Machine Learning (FML) is a technique that can be used in this context to preserve the privacy of sensitive information and provide explainability for the models adopted. Explainability techniques are an essential resource for interpreting the classification performed by the model. In this regard, this study aims to evaluate, through the adoption of specific similarity indices, the robustness and consistency of the explainability algorithms adopted to identify the areas of the images that are decisive for PPE classification. The dataset consists of 1600 real images representing work environments, in which staff are portrayed both with and without Personal Protective Equipment; specifically, there are workers wearing helmets, workers wearing reflective vests, workers wearing both devices and, finally, workers without any PPE. SSIM, VIF and SCC are the most relevant indices involved in the study. In the experimental phase, their mean values stand at 0.99, 0.96 and 0.96 for the intra-client study, and 0.96, 0.91 and 0.71 in the inter-client analysis.

## 1. Introduction

Workplace Safety is a crucial factor in construction and industrial contexts. Data provided by the International Labour Organisation (ILO) show that every year, 2.78 million workers lose their lives due to accidents at work and occupational diseases [1]. There are several causes for this, such as failure to use PPE or incorrect use of PPE, lack of adequate governance and regulations, and misinformation about the importance of health prevention in the workplace. Over the last few years, the topic of workplace safety has also been analysed using Artificial Intelligence (AI) tools. In this vein, the issue of safety in industrial settings using AI techniques has been addressed in several studies. For instance, Isalovic et al. employed Convolutional Neural Networks (CNNs) to classify a dataset comprising twelve different types of PPE. Several key architectures were tested, including Faster R-CNN, MobileNetV2-SSD and YOLOv5; the results show that YOLOv5 achieves the best performance, with a recall rate of 0.61 [2]. Han and Zeng conducted a binary analysis on the detection of items within the image set under examination and demonstrated that the average precision metric stands at 0.92 [3]. Furthermore, anomaly detection in industrial settings has also been addressed through the use of Vision Transformers (ViT), architectures that adapt self-attention mechanisms to computer vision tasks, where each image is broken down into patches and transformed into an embedding capable of preserving the original spatial information. The core of the architecture enables the model to assess the relative importance of each patch compared to the others across the entire image, capturing global relationships and proving more efficient from the very first layer. For these reasons, the work by Han et al. [4] appears extremely interesting: they have in fact proposed an integrated method based on ViT capable of processing spatio-temporal data relating to the monitoring of human activity in the context of surveillance.

Among emerging AI techniques, it is important to note that Federated Machine Learning (FML) has proven useful for analysing images of workers. In fact, in this context, FML enables classification and guarantees the privacy of sensitive information thanks to the process of data decentralisation, whereby the central server updates the weights sent by the local servers on which training takes place. This reduces the risk of sensitive data being exposed. For example, the study conducted by Di Renzo et al. [5] proposes a binary classification relating to images of workers in safety and those who are not. In particular, the adoption of FML through a ViT architecture has helped to achieve an accuracy of 0.77.

Starting from these considerations, in this study, we propose a multi-class analysis (where the classes are helmet, reflective jacket, unsafe worker and safe worker) using an FML approach in which the explainability of the models is guaranteed by the use of heatmaps. The aim of the analysis is to compare the different heatmaps created, including Grad-CAM, attention, Rollout and CLS, and to demonstrate their reliability through similarity indices such as the Structural Similarity Index Measure (SSIM), Visual Information Fidelity (VIF) and Spatial Correlation Coefficients (SCCs). In this context, the article aims to provide tools capable of validating the consistency of the activation maps needed to understand which areas of the images have most influenced the model’s classification. In particular, an intra-model comparison (analysis of different heatmaps on a single model of client) and inter-model comparison (same heatmap for different models of clients) is performed. The inter- and intra-client comparisons demonstrate the consistency of the internal reasoning of each model. The rest of the article is structured as follows: Section 2 describes the method adopted for the experimental analysis. Section 3 describes the experimental analysis and the results relating to the similarity indices obtained by evaluating the different activation maps in the intra-client and inter-client study. Moreover, Section 4 discusses the state of the art of the automatic PPE detection and, finally, in Section 5, the conclusions and future work connected to the explainability techniques and quantitative analysis of PPE detection are discussed.

## 2. The Method

**Figure 1:**
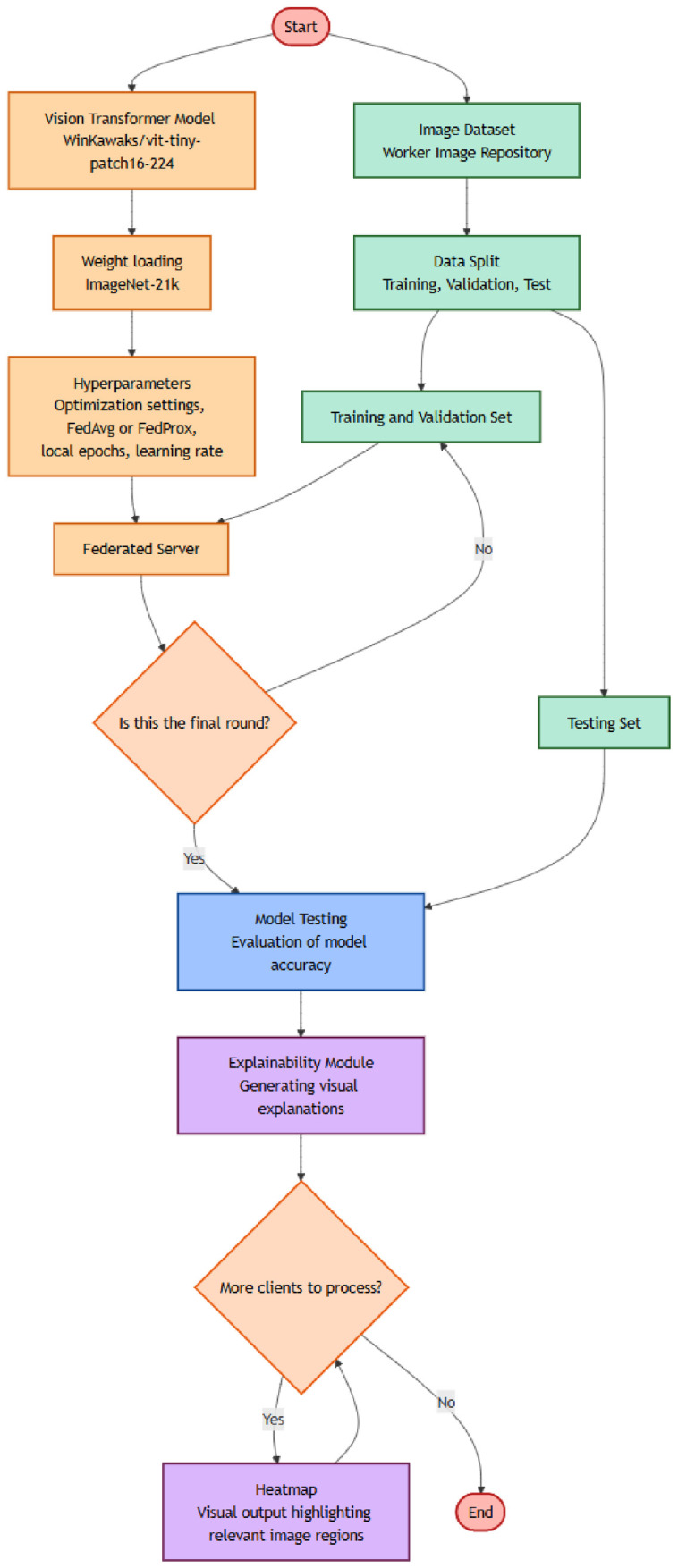
The workflow of the proposed method for explainable and federated PPE classification.

Figure 1 shows the proposed Federated Learning-based method employed for privacy-preserving PPE detection. The process begins with a central server that initializes a global base model and disseminates it to all participating client nodes. Each client operates within a privacy-sensitive workplace environment and acquires images from heterogeneous on-site surveillance systems, which may vary in resolution and device characteristics. To ensure consistency across these decentralized data sources, all images undergo a standardized pre-processing stage that resizes the inputs to 224×224 pixels prior to training. In the proposed configuration, the data is divided among the clients according to an IID (Independently and Identically Distributed) distribution, ensuring that each node analyses a uniform subset. The dataset used for the analysis consists of 1600 images; consequently, each subset comprises 160 images during the local training phase. With regard to the federated model in this paper, we consider the pretrained WinKawaks/vit-tiny-patch16-224 ViT model, freely available for research purposes on the HuggingFace repository (https://huggingface.co/WinKawaks/vit-tiny-patch16-224 (accessed on 26 April 2026). This model processes each image by dividing it into fixed-size 16×16 patches, embedding them into a sequence of tokens, and propagating them through multiple layers of multi-head self-attention. The entire ViT backbone is frozen to preserve its robust, pre-learned visual representations and to prevent overfitting in client environments where training samples may be limited. A lightweight task-specific classification head is appended to the frozen backbone: it consists of a fully connected layer that projects the ViT output into a 128-dimensional latent space, followed by a ReLU activation, a dropout layer with a rate of 0.3 for regularization, and a final linear layer that outputs logits corresponding to the “safe”,“unsafe”, “vest” and “helmet” PPE classes: in particular, `safe’ refers to the group of workers who wear both a helmet and a jacket, whilst ‘unsafe’ refers to workers who do not use any PPE. The `helmet’ class consists solely of workers who wear only a helmet, without any other PPE; similarly, the ‘jacket’ class includes only workers who wear a jacket. Therefore, each group is associated with a single label. This hybrid design leverages the general-purpose feature extraction of the pretrained transformer while enabling fine-tuning for the downstream classification task.

In Listing 1, we provide a code snippet we developed in the Python programming language (3.9.0. version) programming language, which aims to show the ViT model implementation.

**Listing 1.** Code snippet developed by authors related to the exploited ViT model.

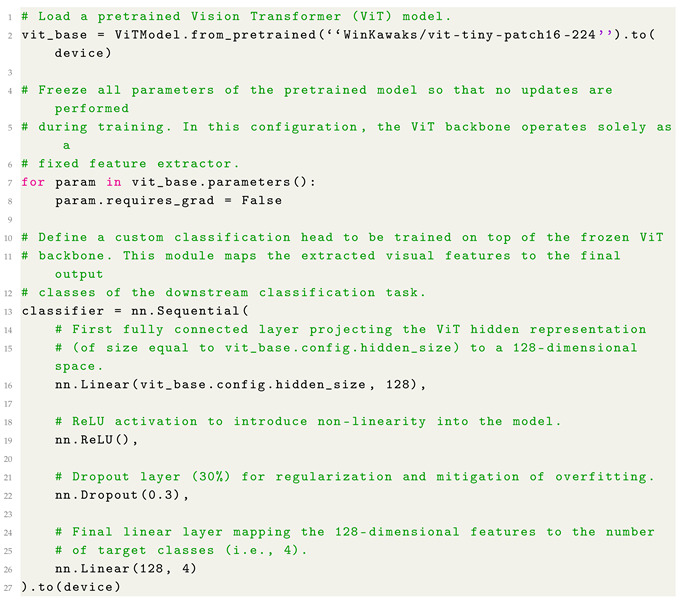



As shown in Listing 1, we employ a Vision Transformer (ViT), a transformer-based architecture specifically designed for image classification tasks. In this work, we make use of the pretrained WinKawaks/vit-tiny-patch16-224 model provided by the Hugging Face transformers library (https://huggingface.co/WinKawaks/vit-tiny-patch16-224 (accessed on 26 April 2026), freely available for research purposes. The choice of a pretrained model, i.e., a model whose weights have been initialized through large-scale image classification tasks, is considered with the aim to ensure that generalizable visual features are already encoded in the proposed model.

Below, we summarize the instructions shown in the Python code snippet reported in Listing 1:

- Feature Extraction with ViT:
  The pretrained ViT model is first loaded and its parameters are frozen, preventing any weight updates during training. In this configuration, the ViT backbone is used strictly as a feature extractor. This approach, known as transfer learning, is particularly effective when the available dataset is limited or when pretrained visual representations are deemed sufficient for the downstream task.
- Custom Classification Head: A custom fully connected (FC) classifier is appended on top of the frozen ViT encoder. This classifier consists of the following components:
  1. A linear layer that maps the ViT hidden representation to a 128-dimensional feature vector.
  2. A ReLU activation function to introduce non-linearity.
  3. A dropout layer with a dropout probability of 0.3 to reduce overfitting.
  4. A final linear layer that projects the 128-dimensional vector to the number of target classes (four in our case).

Once the global model has been broadcast to the clients, each client uses its own locally stored dataset, kept entirely on-device to maintain data confidentiality, with the aim to train the classification head using its local samples. Instead of sharing raw images, clients return only their updated model parameters to the central server. The server then aggregates these updates into an improved global model using either the classical Federated Averaging (FedAvg) algorithm or the Federated Proximal (FedProx) method. FedAvg computes a weighted average of client parameters according to local dataset sizes, while FedProx introduces a proximal constraint to stabilize training under non-IID client distributions by penalizing excessive deviation from the global model.

After aggregation, the refined global model is redistributed to the clients, and this cycle of local training and global updating continues for multiple federated rounds, progressively enhancing predictive performance and generalization across the distributed environment. Upon convergence, the final global model proceeds to an explainability stage in which a set of explainability techniques, i.e., attention, Grad-CAM, CLS and Rollout, is applied to a separate test set. These complementary methods generate heatmaps that highlight the image regions most influential to the model’s decisions, allowing verification that attention is correctly directed toward semantically meaningful PPE components such as helmets and reflective vests. This post-training explainability analysis ensures that the final model remains transparent, auditable, and consistent with safety-critical requirements while fully preserving the privacy of all client-side data throughout the entire workflow.

In the following, we provide a brief explanation related to the explainability techniques we considered.

The first one is the attention mechanism that, in ViTs, allows the model to focus on specific areas of an input image when making predictions. It works by calculating attention scores for each image patch in relation to others, essentially highlighting key regions of the image that influence the decision. These attention scores can then be visualized as attention maps, offering insight into which parts of the image the model prioritizes when making its prediction.

The second one is the Grad-CAM (Gradient-weighted Class Activation Mapping), which, in contrast, helps visualize which regions of an image are most influential in the model’s output. By computing the gradient of the output with respect to the final convolutional layer, Grad-CAM generates a weighted combination of feature maps that can be used to create a heatmap. This heatmap can be overlaid on the original image, visually showing which areas are most significant for the model’s decision.

The third one is the CLS (class token), that plays a critical role in ViT decision-making. This special token, added to the input image’s patch embeddings, captures global information about the image. After passing through the transformer layers, the final representation of this class token is used for classification, and by examining how the class token evolves across layers, we gain a clearer understanding of how the model processes the image and refines its predictions.

The fourth one is the Rollout, i.e., an advanced technique that helps visualize the self-attention maps within a transformer model. It builds on the concept of self-attention but extends it across multiple layers, providing a more comprehensive view of how attention is distributed and how different patches interact with one another. This iterative “rollout” process shows how information flows through the network, helping us to understand the relationships between patches and how the model integrates these dependencies to make its final decision.

The explainability obtained with the adoption of these four different techniques provides a visual understanding with regard to the model decision, i.e., a qualitative analysis related to explainability. With the aim of providing a quantitative analysis of explainability, we provide two categories of metrics that we compute with the aim of evaluating the explainability of the model from a quantitative point of view.

The first category is the intra-model one, referred to as the quantitative analysis related to the single client model, with the aim of evaluating how the different explainability techniques agree on the areas on the images detected as being of interest for a model of a client.

The second category is the inter-model one, referred to as the quantitative analysis related to a different client, with the aim of evaluating how two clients (and, thus, two different models) agree on the areas on the images detected as being of interest for both clients.

**Figure 2:**
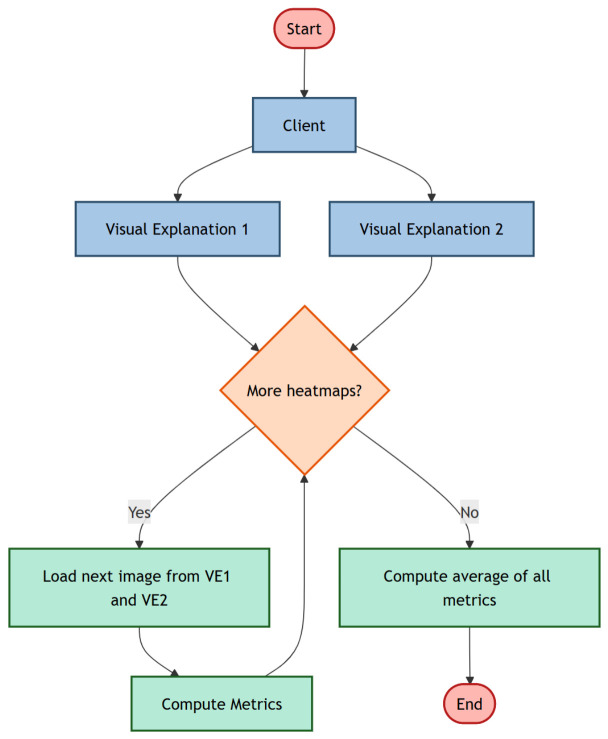
The workflow for the intra-model evaluation.

**Figure 3:**
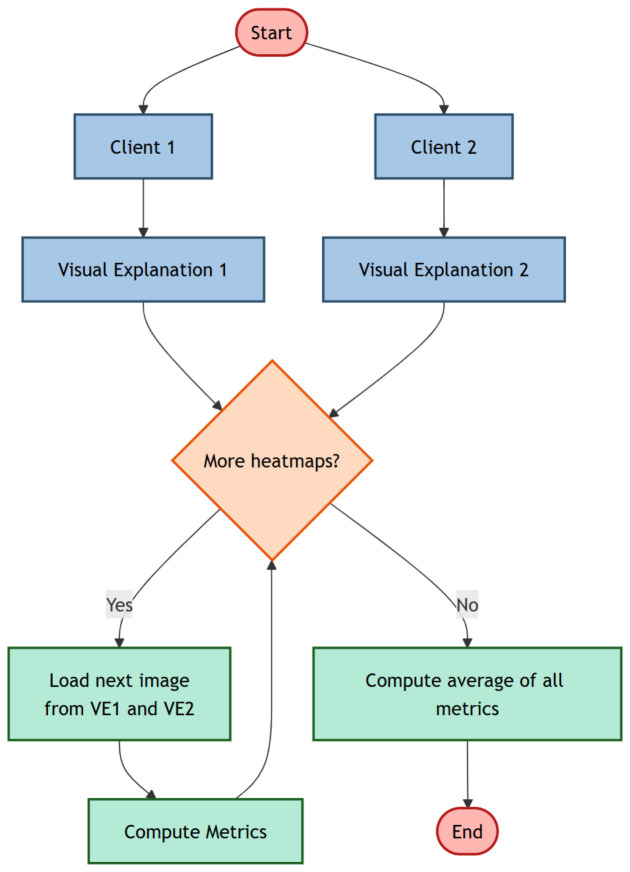
The workflow for the inter-model evaluation.

Once the two categories are defined, Figure 2 and Figure 3 show the workflow for the quantitative analysis for the intra-model and for the inter-model categories, respectively. Thus, after each client produces its explainability outputs using the final global model, we first perform an intra-model evaluation, shown in Figure 2. In this stage, the explainability techniques are applied to the same server-trained model and the same input image. This allows us to compare how different explainability algorithms highlight relevant regions. High spatial overlap between the resulting heatmaps provides evidence of internal consistency in the model’s reasoning, confirming that distinct CAM algorithms converge on similar PPE-related features.

Figure 3 illustrates the inter-model explainability analysis. Here, we apply a single explainability technique to two models obtained from two different clients. This comparison focuses on whether the two federated models attend to the same image regions when making PPE compliance predictions.

In both the categories, i.e., the intra-model and the inter-model, we compute three different metrics (ranging from 0 to 1, where 1 is the best result) between the heatmaps: the Structural Similarity Index Measure (SSIM) and the Visual Information Fidelity (VIF) and Spatial Correlation Coefficient (SCC). SSIM evaluates perceptual similarity between two heatmaps in terms of luminance, contrast, and structural alignment, and it is defined as

$$\mathrm{SSIM}\left(x,y\right)=\frac{\left(2\mu_{x}\mu_{y}+C_{1}\right)\left(2\sigma_{xy}+C_{2}\right)}{\left(\mu_{x}^{2}+\mu_{y}^{2}+C_{1}\right)\left(\sigma_{x}^{2}+\sigma_{y}^{2}+C_{2}\right)},$$

where $\mu_{x}$ and $\mu_{y}$ denote the mean intensities, $\sigma_{x}^{2}$ and $\sigma_{y}^{2}$ the variances, and $\sigma_{xy}$ the cross-covariance of heatmaps *x* and *y*, with $C_{1}$ and $C_{2}$ stabilizing constants. VIF, computed using the implementation vifp from sewar.full_ref, measures the amount of visual information preserved between two heatmaps using a wavelet-based natural scene statistics model:

$$\mathrm{VIF}\left(x,y\right)=\frac{\sum_{i}\log\left(1+\frac{g_{i}^{2}\sigma_{x,i}^{2}}{\sigma_{v,i}^{2}}\right)}{\sum_{i}\log\left(1+\frac{\sigma_{x,i}^{2}}{\sigma_{n,i}^{2}}\right)},$$

where $g_{i}$ is the gain factor between reference and distorted signals, $\sigma_{x,i}^{2}$ and $\sigma_{v,i}^{2}$ represent the signal and visual noise variances, and $\sigma_{n,i}^{2}$ denotes the natural noise variance. Finally, the Spatial Correlation Coefficient (SCC) was introduced, which measures the local spatial correlation between activation maps. This coefficient provides an assessment of the degree of alignment of spatial structures and the distribution of gradients between two heatmaps:

$$SCCx,y=\frac{\sum_{i=1}^{n}\left(x_{i}-\bar{x}\right)\left(y_{j}-\bar{y}\right)}{\sqrt{\sum_{i=1}^{n}{\left(x_{i}-\bar{x}\right)}^{2}}\sqrt{\sum_{i=1}^{n}{\left(y_{i}-\bar{y}\right)}^{2}}}$$

where *x_i_* represents the pixel value in the first reference heatmap, *y_i_* the pixel value in the second reference heatmap, $\bar{x}$ stands for the mean pixel value of the first heatmap, $\bar{y}$ stands for the mean pixel value of the second activation map, and finally *n* represents the total number of pixels.

High SSIM, VIF and SCC values indicate strong agreement between CAM methods on the same model.

By combining intra-model and inter-model comparisons, the idea is to ensure that both the internal reasoning of each model and the consistency across models remain aligned with meaningful and safety-critical PPE features.

In this context, it should be noted that the aim of our work is to analyse the consistency between different explainability techniques and between federated models, rather than merely assessing semantic correctness against an annotated gold standard. In this regard, the SIM, VIF and SCC indices have been chosen as metrics for the quantitative assessment of similarity between explainability techniques on different clients and between different explainability techniques on the same client.

## 3. Experimental Analysis

In this section, we present the experimental analysis we conducted to demonstrate the effectiveness of the proposed method. The analysis aims to evaluate the performance of the model using a technique that compares inter- and intra-model heatmaps using the SSIM and VIF similarity metrics.

### 3.1. Dataset

In this study, we relied on publicly accessible datasets from Kaggle and Roboflow, which include images depicting workers with and without Personal Protective Equipment (PPE). In particular,

- Worker Only Vest Dataset [6].
- PPE and Heavy Machinery Detection Dataset [7].
- Worksite Safety Monitoring Dataset [8].
- Human Detection Dataset [8].

**Figure 4:**
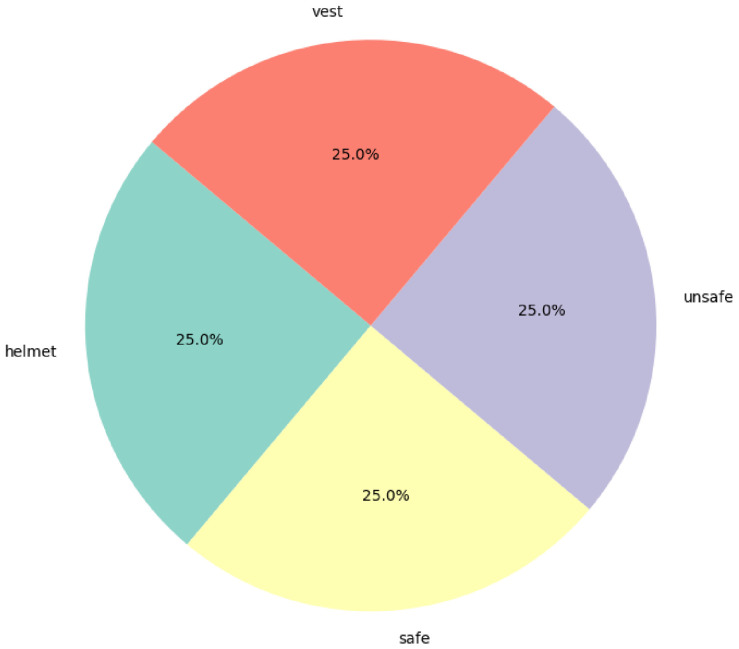
The diagram shows how the images were categorised during the analysis. Each class contains 25% of the total number of images.

Figure 4 shows that the overall dataset consists of 1600 images.

Table 1 below shows the distribution into training validation and testing:

### 3.2. Experimental Settings and Results

**Figure 5:**
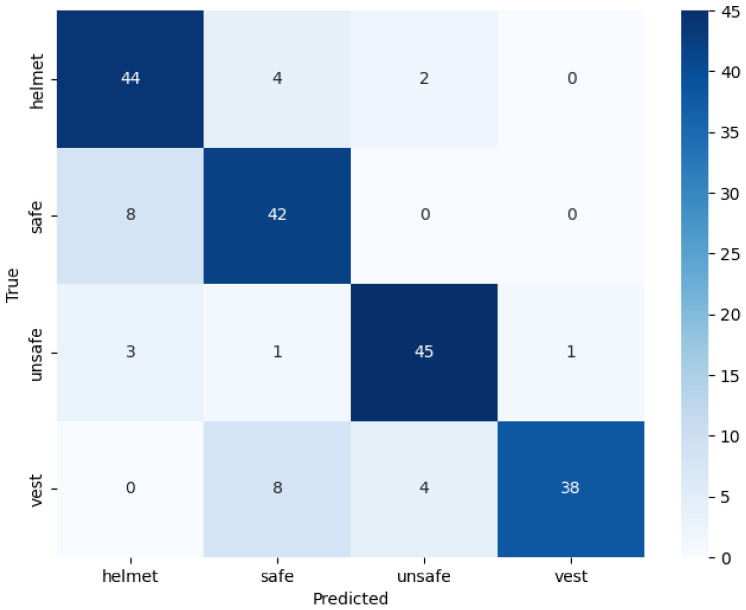
Confusion matrix obtained from Experiment 1.

**Figure 6:**
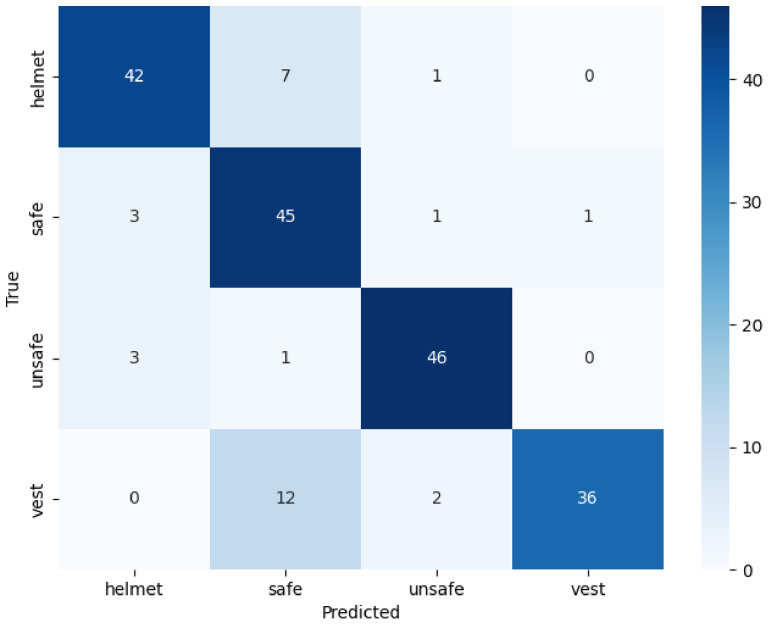
Confusion matrix obtained from Experiment 2.

**Figure 7:**
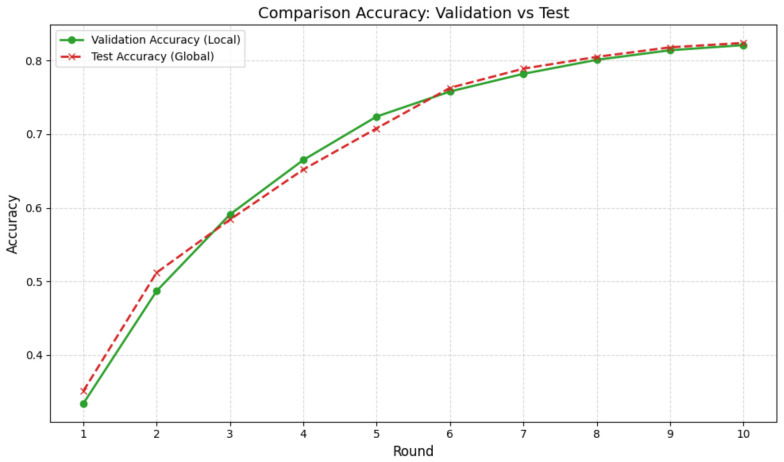
Graph comparing the accuracy trends during the validation and testing phases. These trends demonstrate the model’s ability to generalise, confirming that it is free from overfitting.

Table 2 shows the experimental configurations, using a federated approach, of the models for which the heatmaps were generated, and Table 3 shows their associated performance. In this case, we can see that the accuracy is 0.8450, the recall index hits a max of 0.8450, and the precision hits 0.8551 in the first experiment and 0.8651 in the second experiment; indeed, the latter was selected as the optimal configuration. The confusion matrices shown in Figure 5 and Figure 6 demonstrate the model’s ability to distinguish between the four classes under consideration. Although Experiment 1 shows greater accuracy in distinguishing between the Helmet and Jacket classes, the matrix in Figure 6 for Experiment 2 highlights the better performance on the classes most critical to safety, namely, safe and unsafe. In particular, Experiment 2 correctly labels 45 cases as safe and 46 as unsafe, ensuring greater reliability. This was done to ensure better generalisation, resulting from the doubling of the number of customers involved, and the greater accuracy, which reduces false positives, thereby enabling the production of more consistent heatmaps focused on areas of interest. Furthermore, the high performance achieved with the FedAvg algorithm indicates that the system is stable and scalable, rendering the adoption of more complex regularisation techniques such as FedProx unnecessary. The graph in Figure 7 shows the trend in accuracy during the testing phase and the validation phase. It can be seen that the values in the testing phase are comparable to those in the validation phase. This ensures the absence of overfitting and good generalisability. The results obtained show that the set of parameters adopted in the experimental phase allows the development of a sensitive and accurate system for the classification of the labels under examination. For these reasons, the study presented involved the use of heatmaps, such as Grad-CAM, attention, CLS and Rollout Heatmaps, to identify areas of greatest interest. Based on the experiments carried out, heatmaps were created for each participating client. The explainability of the results obtained and the robustness of the model created were evaluated and confirmed by the introduction of quantitative statistical indices on the maps generated. We applied min–max normalization to the explainability heatmaps to rescale the activation value [0, 1] range. We consider this normalization because it ensures consistent contrast across samples, allowing for a reliable comparison of salient regions highlighted by the proposed ViT model. In fact, it is believed that the quantitative analysis of heatmaps is an indispensable element for comparing the hotspot detection capabilities of each interpretability algorithm. The overlap of the generated heatmaps and the results obtained for the SSIM, VIF and SCC indices show that different explainability algorithms identify similar and hot regions.

**Figure 8:**
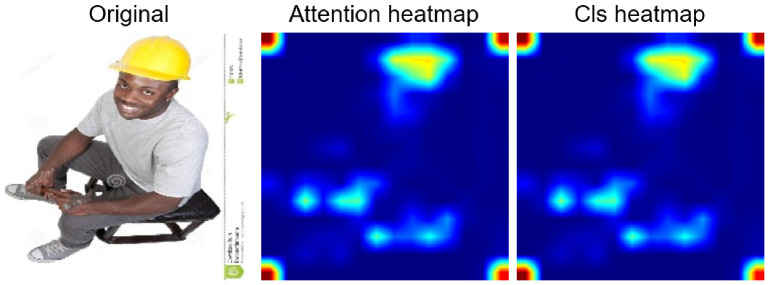
Comparison of attention CLS heatmaps for client 3. The hot areas correctly identify the position of the helmet in the original image.

**Figure 9:**
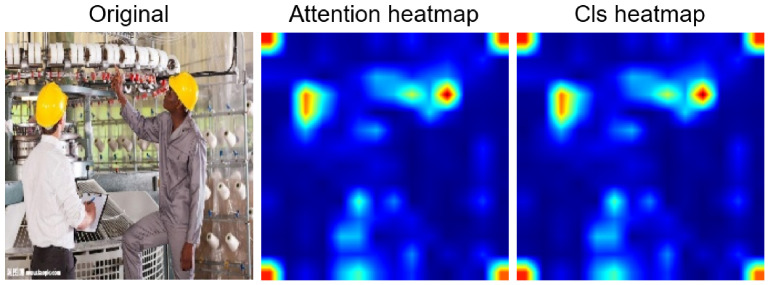
Comparison of attention CLS heatmaps for client 7. The two hot areas in the centre of the heatmap indicate that the two workers in the original image are wearing helmets.

**Figure 10:**
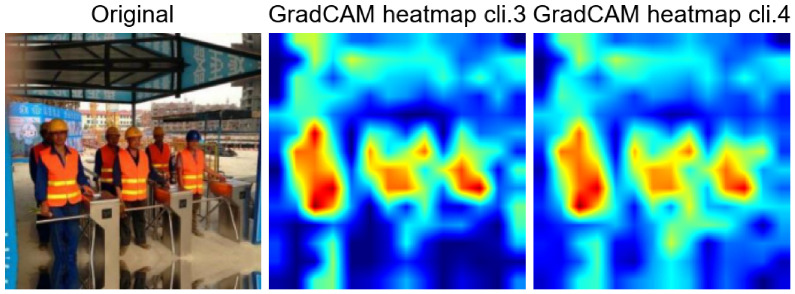
Comparison of Grad-CAMs between clients 3 and 4. The hot areas indicate the presence of helmets and vests associated with safe workers.

**Figure 11:**
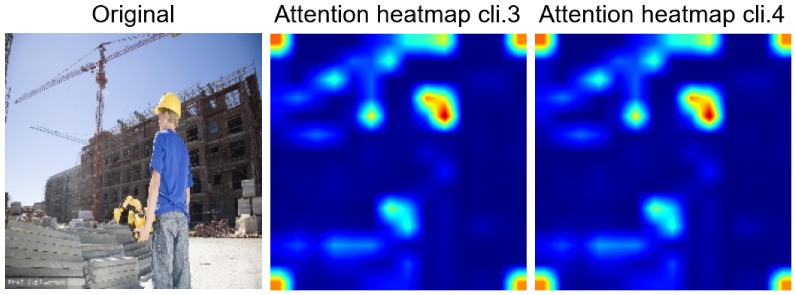
Comparison of attention heatmaps between clients 3 and 4. The central hot region refers to the helmet worn by the worker in the original image.

**Figure 12:**
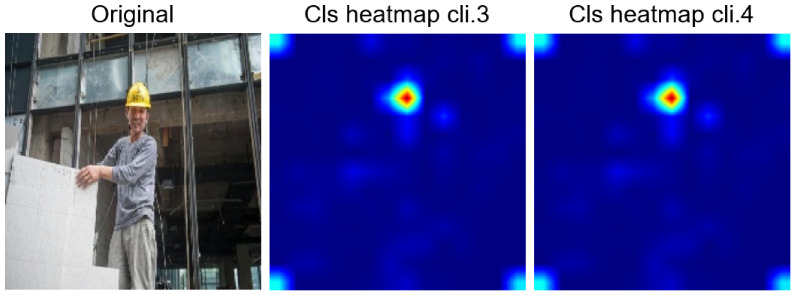
Comparison of CLS heatmaps between clients 3 and 4. In this case, the only area highlighted on the heatmap corresponds to the area where the worker is wearing the helmet.

**Figure 13:**
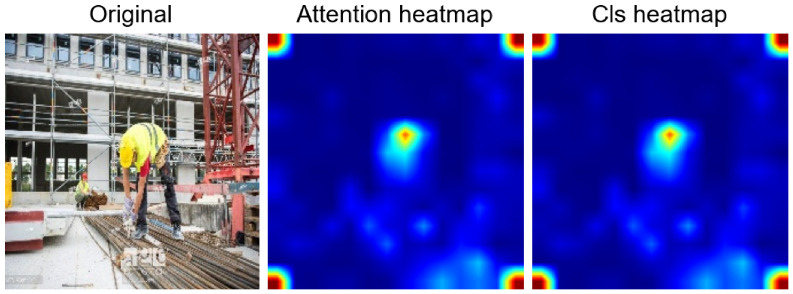
Comparison of attention and CLS heatmaps between clients 3 and 4. The central area highlighted on the heatmap corresponds to the presence of PPE the original image.

**Figure 14:**
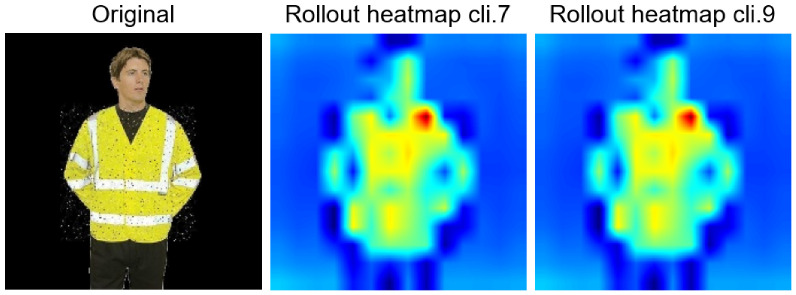
Comparison of Rollout heatmaps between clients 7 and 9. The heatmap is only activated in the area corresponding to the presence of vest in the image.

The study consisted of two main phases: the first phase involved an intra-model analysis, where all the heatmaps generated by the explainability algorithms were compared; the second phase coincided with the inter-model analysis, in which, for each experiment, the heatmaps generated from the different client models considered were compared. Table 4 below shows the best results for the quantitative indices considered after the overlap of the heatmaps for the intra-model study. Table 5 shows the results of the inter-model analysis. The results presented in Table 4 and Table 5, obtained from the quantitative evaluation, highlight the high consistency of the explanations provided by the model. In particular, in the intra-client study, the similarity indices reach high average values: a value of 0.99 is obtained for the SSIM and 0.96 for both the VIF and the SCC. These data quantitatively demonstrate how different explainability techniques, applied to the same model, converge almost perfectly on the same regions of interest. Similarly, when analysing the inter-client configuration, slightly lower values are observed due to the comparison of models belonging to different clients; however, the results remain high. The average SSIM value reaches 0.96 and the VIF 0.91. These values highlight that, despite differences in local training, the explainability techniques continue to consistently focus on the same regions of interest. The observed drop in the SCC index in the inter-client configuration is due to its high sensitivity to point-specific variations. This allows us to understand that the region of interest remains consistent across the different clients, but the distribution of pixels in the heatmaps undergoes morphological variations due to the specificities of local training. The SSIM, VIF and SCC indices have a maximum value of 1. This demonstrates the learning effectiveness of the federated model; the areas (in red) of interest identified by the explainability algorithms show a high degree of overlap and good consistency in terms of brightness, contrast and texture. The different heatmaps are therefore consistent with each other in terms of brightness, contrast and texture in both the inter-model and intra-model analyses. For these reasons, the contribution to the classification by each client converges with that of the others; this result is demonstrated by the fact that the hot areas of interest are completely overlapping. The heatmaps shown in Figure 8, Figure 9, Figure 10, Figure 11, Figure 12, Figure 13 and Figure 14 provide a visual and qualitative representation of the results achieved by the similarity indices.

## 4. Related Work

The progressive increase in accidents in the workplace and the consequent monitoring of the correct use of PPE has led to the development of AI tools applied to the context in question. Several studies have investigated PPE detection using deep learning techniques, highlighting their applicability in real-world industrial environments [1,9,10,11,12,13].

The surveillance and monitoring of human actions remain a significant challenge for anomaly detection in industrial settings. For this reason, Han et al. [4], in their work, proposed an integrated ViT-based method that processes spatio-temporal data, where the position-encoding module organises non-sequential information, whilst the transformer encoder efficiently compresses the features of sequential data to improve computational speed. Human activity recognition is achieved using a multi-layer perceptron (MLP) classifier.

In this context, the authors of [1] proposed a real-time PPE detection system. Their study supports the effectiveness and importance of DL models in improving safety on construction sites through automated verification of the correct use of safety equipment. Additional contributions have shown the benefit of large-scale curated datasets for robust PPE recognition in diverse occupational environments [6,7,8,14].

Han and Zeng also addressed the issue of detecting safety helmets on construction sites using Deep Learning techniques. In this case, a dataset consisting of images of construction sites sourced from the internet was used; the study employed YOLOv5 as the main algorithm and incorporated a four-level detection method. The main evaluation metric was the mean average precision (mAP) [3]. The study also focused on detecting whether workers were wearing helmets. The results of this study achieved an mAP of 0.92, which is better than using YOLOv5 alone, which achieved 0.86. However, the work described does not guarantee the privacy and security of sensitive data contained in the image datasets, nor does it provide an explainability technique capable of demonstrating the system’s robustness through visual representations of the areas that are critical for classification.

The use of CNNs in PPE classification was addressed by Isalovic et al. [2], who tested various architectures including Faster R-CNN, MobileNetV2-SSD and YOLOv5. The proposed pipeline integrates the estimation of the area of interest on the head with a PPE detection system, using a dataset containing 12 different types of PPE. The results show that YOLOv5 achieves superior performance with a recall rate of 0.61.

A safety analysis, based mainly on the classification of images of workers on construction sites, was also conducted using FML, which proved necessary for privacy protection. For example, the FedSH framework considers a solution enabling collaboration across multiple worksites without centralising sensitive data, ensuring both scalability and privacy [15,16,17,18].

The superiority of FL over traditional learning techniques has also been demonstrated by Makris et al., who developed an innovative approach integrating FL with the YOLOv8 architecture for advanced PPE object detection [19]. Their results indicate that FL offers strong performance while complying with strict requirements for privacy and data protection.

Although FML techniques are effective for the task at hand, model explainability remains crucial for understanding automated decisions. In this context, explainability techniques such as Grad-CAM [20,21] and transformer attention-based methods [22,23,24] play a central role in ensuring transparency in monitoring systems.

For instance, the method proposed by Di Renzo et al. proposes a federated, privacy-preserving PPE detection system enriched with Grad-CAM visual explanations [5,25]. This integration helps reveal the discriminative image regions that most influence the model’s decisions, improving trust and safety in workplace environments.

## 5. Conclusions and Future Work

The results obtained from the experiments demonstrate the effectiveness and suitability of the federated model for detecting PPEs in the building and industrial contexts. The values assumed by the SSIM and VIF similarity indices confirm the decision-making consistency of the explainability algorithms adopted in the correct identification of hotspots in the images and confirm the decision-making importance of the clients involved in predicting the labels examined. These indices allow for the assessment of the consistency of the localisation regions identified by the local models. As future work, it would be useful to include additional interpretability algorithms in order to evaluate their robustness on intra- and inter-model analysis. Furthermore, the integration of annotated masks and bounding boxes represents a necessary step towards transforming explainability from a qualitative analysis into rigorous quantitative validation. This is because SSIM, VIF and SCC ensure consistency among explanations, and therefore, only a direct comparison with the ‘ground truth’ (i.e., manual annotations) can demonstrate that the federated model has captured the distinctive semantic features of PPE. This approach would enable an increase in the model’s accuracy.

## Figures and Tables

**Table 1:** Dataset breakdown into train, validation and test.

Set	Safe	Unsafe	Helmet	Vest	Total
Train set	300	300	300	300	1200
Validation set	50	50	50	50	200
Test set	50	50	50	50	200

**Table 2:** Tested configurations.

Exp	Total Clients	Epochs	Nr. Rounds	Learning Rate	Batch Size	Aggregation
1	5	100	10	0.001	32	FedProx
2	10	200	10	0.001	32	FedAvg

**Table 3:** Performance obtained for each experiment.

Exp	Loss	Accuracy	Precision	Recall	F1-Score	AUC
1	0.4530	0.8450	0.8551	0.8450	0.8458	0.9680
2	0.4873	0.8450	0.8651	0.8450	0.8468	0.9644

**Table 4:** Best results obtained by the intra-client analysis.

Client 1	Client 2	Heatmap 1	Heatmap 2	SSIM	VIF	SCC
3	3	Attention	CLS	1	1	0.96
7	7	Attention	CLS	0.98	0.72	0.96
intra-client mean values				0.99	0.96	0.96

**Table 5:** Best results obtained by the inter-client analysis.

Client 1	Client 2	Heatmap 1	Heatmap 2	SSIM	VIF	SCC
3	4	Attention	Attention	1	1	0.65
3	4	Grad-CAM	Grad-CAM	0.79	0.54	0.40
3	4	CLS	CLS	1	1	0.65
3	4	Attention	CLS	1	1	0.99
7	9	Rollout	Rollout	1	1	0.99
inter-client mean values				0.96	0.91	0.71

## Data Availability

The original contributions presented in this study are included in the article. Further inquiries can be directed to the corresponding authors.
